# Glucose transporters GLUT1, GLUT3, and GLUT4 have different effects on osteoblast proliferation and metabolism

**DOI:** 10.3389/fphys.2022.1035516

**Published:** 2022-11-29

**Authors:** Milja Arponen, Niki Jalava, Nicko Widjaja, Kaisa K. Ivaska

**Affiliations:** Institute of Biomedicine, University of Turku, Turku, Finland

**Keywords:** glucose transporter (GLUT), osteoblast, proliferation, bone marrow stromal cell (BMSC), glucose metabolism

## Abstract

Bone is an active tissue that undergoes constant remodeling. Bone formation requires energy and one of the energy sources of bone-forming osteoblasts is glucose, which is transported inside the cells *via* glucose transporters. However, the role of class I glucose transporters in the differentiation and metabolism of osteoblasts and their precursors, bone marrow mesenchymal stromal cells (BMSCs) remains inconclusive. Our aim was to characterize the expression and contribution of main class I glucose transporters, GLUT1, GLUT3, and GLUT4, during osteoblast proliferation and differentiation. To investigate the role of each GLUT, we downregulated GLUTs with siRNA technology in primary rat BMSCs. Live-cell imaging and RNA-seq analysis was used to evaluate downstream pathways in silenced osteoblasts. Glucose transporters GLUT1, GLUT3, and GLUT4 had distinct expression patterns in osteoblasts. GLUT1 was abundant in BMSCs, but rapidly and significantly downregulated during osteoblast differentiation by up to 80% (*p* < 0.001). Similar downregulation was observed for GLUT4 (*p* < 0.001). In contrast**,** expression levels of GLUT3 remained stable during differentiation. Osteoblasts lacked GLUT2. Silencing of GLUT4 resulted in a significant decrease in proliferation and differentiation of preosteoblasts (*p* < 0.001) and several pathways related to carbohydrate metabolism and cell signaling were suppressed. However, silencing of GLUT3 resulted in increased proliferation (*p* < 0.001), despite suppression of several pathways involved in cellular metabolism, biosynthesis and actin organization. Silencing of GLUT1 had no effect on proliferation and less changes in the transcriptome. RNA-seq dataset further revealed that osteoblasts express also class II and III glucose transporters, except for GLUT7. In conclusion, GLUT1, -3 and -4 may all contribute to glucose uptake in differentiating osteoblasts. GLUT4 expression was clearly required for osteoblast proliferation and differentiation. GLUT1 appears to be abundant in early precursors, but stable expression of GLUT3 suggest also a role for GLUT3 in osteoblasts. Presence of other GLUT members may further contribute to fine-tuning of glucose uptake. Together, glucose uptake in osteoblast lineage appears to rely on several glucose transporters to ensure sufficient energy for new bone formation.

## Introduction

Osteoblasts are bone-forming cells which produce a large amount of extracellular matrix and have high demand for both energy and building blocks during bone formation. Glucose is a central source of energy and carbon for most mammalian cells, and accumulating evidence suggest that osteoblast differentiation and bone formation are dependent on glucose utilization (Karner and Long, 2018). Emerging data on the interplay between energy metabolism and the skeleton, and the role of the skeleton as an endocrine organ, highlight the importance of understanding glucose handling and bioenergetics properties of bone cells (Lee et al., 2017; Cipriani et al., 2020).

In most mammalian cells, cellular uptake of glucose and other hexoses is mediated by specific membrane-associated carrier proteins, namely facilitated glucose transporters (GLUTs) or the sodium glucose cotransporters (SGLTs). To date, fourteen members of the extended GLUT family, encoded by the *Slc2A* gene family, are known to be expressed in human (Mueckler and Thorens, 2013). They are classified into three groups, according to sequence similarities and characteristics. Class I comprise of well-characterized facilitative glucose transporters from GLUT1 to GLUT4, class II fructose-specific transporter GLUT5 and related proteins (GLUT7, −9 and −11) and class III less characterized homologues (GLUT6, −8, −10, and −12). Glucose transporters exhibit tissue-specific expression patterns, different affinities to glucose and different sensitivities to stimuli, such as insulin, providing the basis for the fine-tuning of glucose uptake to cellular requirements (Joost and Thorens, 2001; Mueckler and Thorens, 2013).

Expression of one or more GLUTs have been reported in osteoblast-like cell lines (Thomas et al., 1996; Zoidis et al., 2011; Pacicca et al., 2019), primary mouse osteoblasts (Wei et al., 2015; Li et al., 2016; Ohnishi et al., 2020) and osteoclasts (Indo et al., 2013), rat calvarial bone (Zoidis et al., 2011) and in human intervertebral discs (Richardson et al., 2008) and chondrocytes (Mobasheri et al., 2002). Both GLUT1 and GLUT3 have been detected in rat UMR-106 osteosarcoma cells (Thomas et al., 1996) and immortalized rat osteoblast cell line PyMS (Zoidis et al., 2011), while the presence of only GLUT1 has been reported in human osteosarcoma cell lines MG-63, Saos-2, and U-2 OS (Cifuentes et al., 2011), and in murine MC3T3-E1 and ST2 cell lines (Takeno et al., 2018). Glucose uptake is confounded by altered glucose handling due to neoplastic changes and it may be difficult to extrapolate findings from cancerous cells to normal physiology. Attempts to explain glucose transport mechanisms in primary osteoblasts have been somewhat contradictory. Wei et al. (2015) showed that GLUT1 was predominant in mouse calvarial osteoblast cultures and it was expressed at least two orders of magnitude higher than any other class I glucose transporter. Li et al. (2016) reported expressions of GLUT1, GLUT3, and GLUT4 in undifferentiated neonatal calvarial osteoblast cultures and expression of GLUT4 further increased during osteoblast differentiation. Taken together, one or more passive GLUT transporters appear to be required for osteoblast function.

In this study, we characterized the expression of main class 1 glucose transporters, GLUT1, GLUT3, and GLUT4, during osteoblast proliferation and differentiation. We used rat bone marrow-derived mesenchymal stromal cells (BMSCs) and differentiated them into bone-forming osteoblasts *in vitro*. To investigate the role of GLUT1, GLUT3 and GLUT4 in osteoblast proliferation, differentiation and metabolic pathways we downregulated each GLUT using siRNA-mediated silencing and performed transcriptomic analysis. Further, we profiled the cells for the presence of other isoforms, from GLUT5 to GLUT12, with the exception of GLUT11 that is not expressed in rat cells (Scheepers et al., 2005).

## Materials and methods

### Cell culture

Bone marrow cells were isolated from the long bones of 3–4 week-old Sprague-Dawley female rats. In brief, epiphyses of long bones were cut and bone marrow was flushed with 22 G needle. Bone marrow mesenchymal stromal cell population was enriched by plastic adherence for 48 h and adherent cells were cultured for 4 to 6 days. We used αMEM medium (Gibco, Thermo Fisher Scientific, Inc., United States), supplemented with fetal bovine serum (FBS, 15%, Gibco), HEPES (10 mM, Gibco), GlutaMAX™ (2 mM, Gibco), Penicillin-Streptomycin (100 U/ml, 100 μg/ml, Gibco), Amphotericin B (2.5 μg/ml, Gibco) and dexamethasone (10^−8^ M, Sigma, United States).

BMSCs were then collected with 0.05% trypsin-EDTA (Gibco) and seeded as described. BMSCs were differentiated to osteoblasts using osteoblast induction medium (OM) containing αMEM medium supplemented with FBS (10%), GlutaMAX™ (2 mM), Penicillin-Streptomycin (100 U/ml, 100 μg/ml), sodium β-glycerophosphate (10 mM, Fluka BioChemika) and l-ascorbic acid 2-phosphate (70 μg/ml Sigma, Missouri, United States). Medium was changed every 2 days.

### RNA isolation and quantitative RT-PCR

mRNA expression was analyzed with quantitative RT-PCR. Cells were seeded in 4–6 identical replicates on six well plates with 160,000 cells per microwell plate for undifferentiated BMSCs used as a control and 80,000 cells for osteoblast differentiation. Undifferentiated BMSCs were collected after 24 h of culturing without osteogenic supplements and differentiated cells at days 2, 4, 6, 8, 10, and 12. Total RNA was isolated with either RNeasy Mini RNA Isolation Kit (Qiagen, Germany) or Macherey-Nagel™ Nucleospin™ RNA plus kit (Macherey-Nagel™, Germany) and the concentration was analyzed with NanoDropND-1000 device (NanoDrop Technologies, United States). 0.5–1 µg of RNA was reverse transcribed to cDNA using 5 µM Oligo-dT mRNA-primer (NewEngland BioLabs, United States) and Maxima RT enzyme (Thermo Fisher Scientific). For quantative RT-PCR, we used 100 ng of cDNA and Dynamo HS SYBR green (Thermo Fisher Scientific) with CFX96 RealTime system C1000 Thermal cycler (BioRad, United States). Osteoblast differentiation was evaluated by the expression of alkaline phosphatase (*Alp1*), Runt-related transcription factor 2 (*Runx2*) and osteocalcin (*Bglap*). Primer sequences (purchased from Oligomer, Finland or Integrated DNA Technologies, Belgium) are provided in Supplementary Table S1. The specificity of amplification was checked with gel electrophoresis and RNA isolated from rat tissues (Rat Tissue Total RNA Panel, AMS Biotechnology (Europe), United Kingdom) were used as positive controls (data not shown). The data was analyzed by ΔΔCT-method (Livak and Schmittgen, 2001) and mRNA expression of Cyclophilin B (*Ppib*) was used to normalize the data (Pachot et al., 2004; Kähkönen et al., 2018), which is presented as relative to reference sample. The expression level of *Ppib* remained stable throughout experiments regardless of differentiation or treatment (data not shown).

### Transfection

The siRNA constructs for silencing of GLUT1 (siGLUT1, SR503164), GLUT3 (siGLUT3, SR502308), GLUT4 (siGLUT4, SR515187) and universal scramble control (Control, SR30004), were purchased from OriGene Technologies (United States). Constructs include gene specific 27mer siRNA duplexes, three different duplexes for each GLUT. siTRAN 2.0 (Origene) was used as transfection reagent. Transfection was done according to manufacturer’s instructions. In brief, BMSCs were seeded 150,000 cells per well on six well plates in quadruplicates and allowed to adhere to the wells for 24 h. Medium was changed to transfection-medium containing 10 nM of individual siRNA. Transfections were differentiated for up to 18 h. Fluorescent-labelled transfection control siRNA duplex (Trilencer-27, 10 nM, OriGene) was used to confirm transfection efficiency (Supplementary Figure S1). After transfection, medium was changed to OM medium and cells were cultured for up to 6 days. Cells were harvested for RT-qPCR analysis at days 2, 4, and 6 of differentiation.

### Cell proliferation and viability

Cell proliferation was analyzed by IncuCyte^®^ S3 Live-Cell Analysis System (Essen BioScience, United States). In brief, 18,000 cells were seeded on 48 well plates in four replicates and let to adhere to the wells for 24 h. Cells were transfected as described and let to grow for additional 5 days in OM in IncuCyte^®^ S3. Cells were imaged at 2 h intervals and cell confluency (%) was assessed at each timepoint with user-defined settings. Cell viability was assessed at the end of the culture using alamarBlue^®^ Cell Viability Reagent (Thermo Fisher Scientific) according to manufacturer’s instructions. In brief, cells were incubated in culture medium containing of alamarBlue^®^ -reagent (1/10) for 30–45 min. Fluorescence intensity (excitation/emission 560/590 nm) was measured with EnSight Multimode Plate Reader (PerkinElmer).

### Evaluation of osteoblast differentiation

Osteoblast differentiation was assessed by measuring mRNA levels of *Alpl* and *Bglap* by qPCR as described above. In addition, we measured enzymatic activity of ALP in cell lysates at endpoint. Cells were collected in lysis buffer (50 mM Tris-HCl, 0.1% Triton X-100, 0.9% NaCl, pH 7.6) and ALP activity was measured using 0.15 nM PNPP (0.1 M Tris, 1 mM MgCl_2_, pH 10) as substrate. After 45 min incubation, enzymatic reaction was stopped with 1 M NaOH and absorbance was measured at 405 nm with EnSight Multimode Plate Reader (PerkinElmer, United States). Total protein was measured using Bradford protein assay (Biorad) according to manufacturer’s instruction. Specific ALP activity is presented as A405 (absorbance units, AU) per protein (mg/ml).

### Immunofluorescence

Immunofluorescence staining was performed as described before (Arponen et al., 2020) with following modifications. BMSCs were seeded on coverslips with density of 10,000 cells and let to attach for 24 h. To stain committed osteoblasts, BMSCs were seeded on coverslips and differentiated in OM for 6 days. To stain siRNA-silenced cells, 30,000 cells were plated on coverslips, treated with siRNAs and grown in OM for 96 h (due to downregulation of mRNA levels already at 48 h). Cells were then fixed with 4% paraformaldehyde for 15 min, permeabilized with 0.05% Triton X-100 for 5 min, blocked with 10% normal goat serum (Abcam, United Kingdom) for 1 h at RT and stained with primary antibodies overnight at +4°C. Unless otherwise specified, the following antibodies were acquired from Abcam and reconstituted in 3% w/v bovine serum albumin. Cells were stained with rabbit anti-CD44 (0.2 μg/ml, ab157107), rabbit anti-CD45 (Merck, United States; SAB4502541, 10 μg/ml), mouse anti-CD90 (ab225, 1 μg/ml), mouse anti-GLUT1 (ab40084, 1:1000), rabbit anti-GLUT3 (ab41525, 6.8 μg/ml), rabbit anti-GLUT4 (ab654, 1:1000) and mouse anti-osteocalcin (HyTest, Finland; 4OC85, clone, 2H9 1 μg/ml). For the secondary incubation, cells were stained with Alexa Fluor^®^ 488-conjugated goat anti-rabbit IgG (ab150077, 2 μg/ml) and Alexa Fluor^®^ 594-conjugated goat anti-mouse IgG (ab150116, 2 μg/ml) for 1 h at RT. Cells were washed in 0.1% Tween-20 three times between incubations. Samples were mounted on microscopic slides with Vectashield Antifade containing 1.5 μg/ml DAPI (Vector Laboratories, United States). Cells were imaged with Zeiss Axio Imager 1 (Zeiss, Germany) with standardized settings maintained throughout each experiment. Images were processed in Zen Blue 2 (Zeiss, Germany) and ImageJ 1.53c (Schneider et al., 2012), and pseudo-colored as cyan (Alexa Fluor®- 488 and 594) and magenta (DAPI).

### RNA sequencing

For the RNA sequencing (RNA-seq) analysis, BMSCs were plated in triplicates in 6-well plates at density of 200,000 cells per well. Cells were treated with siRNAs as described and cultured at OM for 48 h. RNA was extracted and samples were stored at −80 °C until analysis.

The mRNA library preparation, RNA-seq, and bioinformatic analysis was purchased from Novogene, Ltd. (Cambridge, United Kingdom). In brief, RNA quality and integrity was first evaluated with BioAnalyser Agilent 2,300 system. mRNA library suitable for RNA-seq was prepared *via* poly-A enrichment. Samples were then sequenced using Illumina NovaSeq platform (Illumina NovaSeq 6,000 sequencing system) using paired-end 150 bp strategy and 20 M reads per sample.

The raw reads were subjected to quality control in which the error rate, GC content, and read quality were evaluated and consequently bad quality reads were filtered. For the read alignment, HISAT2 algorithm was used to map the reads to *rattus norvegicus* reference genome (Rnor_6.0). Alignment data was used for calculation of gene expression levels based on read counts. Differential expression analysis was performed using the DESeq2 R-package (Anders and Huber, 2010) and the resulting *p*-values were adjusted for false discovery rate using the Benjamini–Hochberg multiple testing adjustment procedure (Benjamini and Hochberg, 1995). Functional analysis of Differentially expressed (DE) genes was done by common pathway enrichment analysis referencing to Gene Ontology database using clusterProfiler software (Yu et al., 2012). Adjusted *p*-values <0.05 were considered statistically significant throughout bioinformatic analyses.

### Statistical analysis

Experiments were repeated two to three times and representative data and images are presented. Data is presented as means with standard deviations (SD), unless otherwise stated. Data was first subjected to Shapiro-Wilk’s normality test and Bartlett’s variance test. Comparisons between groups were tested with two-tailed Student’s *t*-test or analysis of variance (ANOVA) with either Dunnet’s or Tukey’s post hoc tests (normally distributed) or Kruskal–Wallis test (nonparametric). When comparing ranks, Mann Whitney U test was used. Nominal significance was considered with *p* < 0.05. Statistical analysis was performed using GraphPad Prism software (8.1.2, GraphPad Software, United States).

## Results

### Various glucose transporters are expressed in osteoblast lineage

**FIGURE 1:**
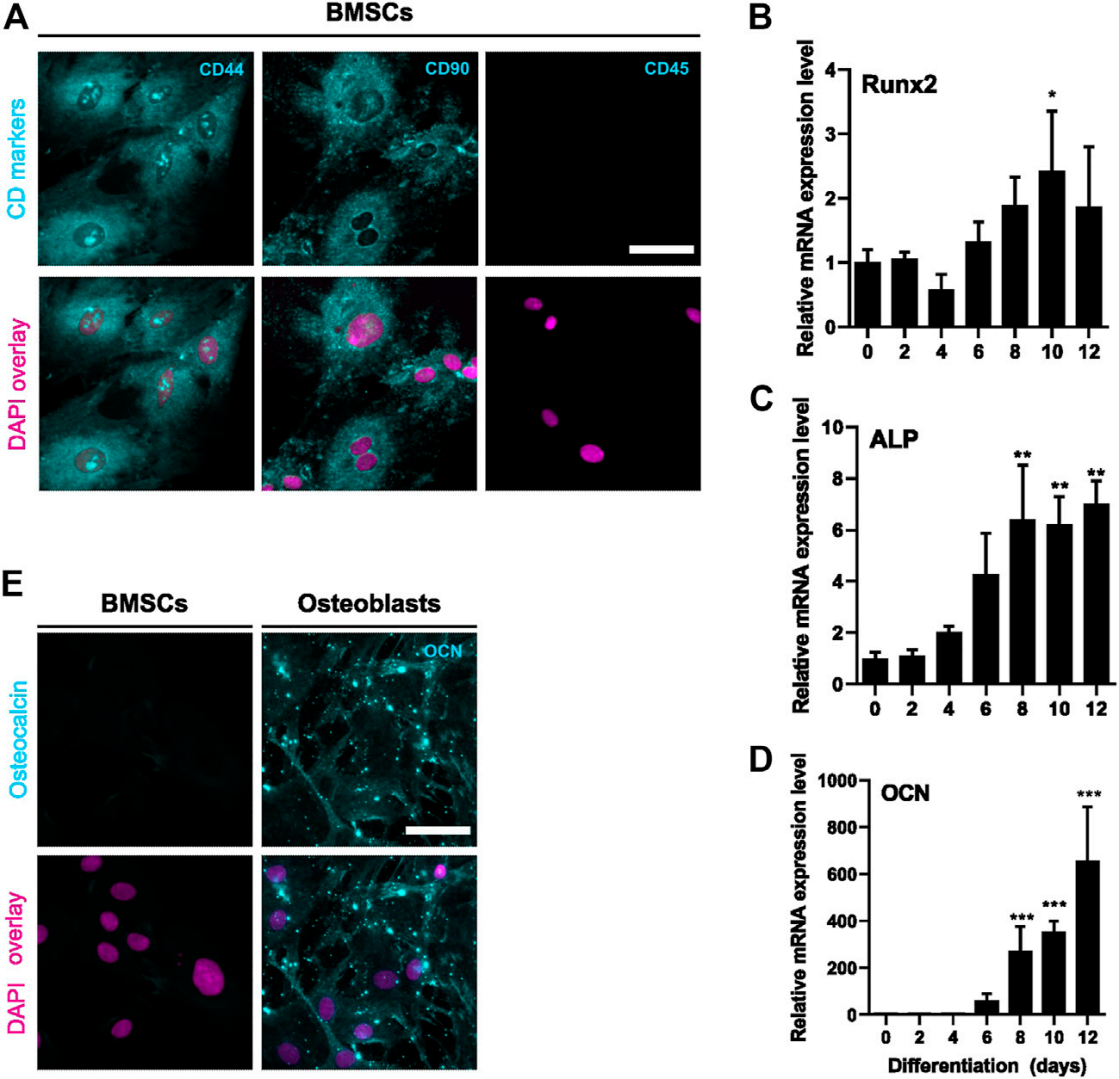
Characterization of BMSC and osteoblastic differentiation. BMSCs were stained for mesenchymal (CD44 and CD90) and hematopoietic (CD45) cell surface markers **(A)**. Protein is shown with cyan and nucleus with magenta. BMSCs were differentiated to osteoblasts for 12 days and characterized for mRNA levels of Runx2 **(B)**, ALP **(C)** and OCN **(D)** (mean ± SD, *N* = 4–6). BMSCs were used as a control (differentiation day 0). Cyclophilin B was used to normalize the mRNA levels. Statistical significances are shown as *p < 0.05, **p < 0.01 and ***p < 0.001 (One way ANOVA). Cultures were repeated 3 times and representative images are shown. BMSCs and day 6 osteoblasts were stained for osteocalcin (cyan) and nuclei (magenta) **(E)**. Scale bar: 50 μm.

We first validated the differentiation of rat BMSCs to bone-forming osteoblasts *in vitro*. BMSCs were positive for mesenchymal markers CD44 and CD90 and negative for hematopoietic marker CD45 (Figure 1A). Commitment to osteoblast lineage was verified by the expressions of Runx2, alkaline phosphatase (ALP) and osteocalcin (OCN). During differentiation, the expression of transcription factor Runx2 increased by approximately 2-fold (*p* = 0.02, Figure 1B) on day 10. Expression of ALP was increased at day 8 (*p* = 0.005) and further up to 7-fold after 12 days (*p* = 0.003, Figure 1C). Osteoblast-specific OCN was also significantly increased from day 8 onwards (*p* = 0.0009) and continued to increase up to 700-fold at 12 days (*p* < 0.0001, Figure 1D). Immunostaining verified the upregulation of OCN protein during osteoblast differentiation (Figure 1E).

**FIGURE 2:**
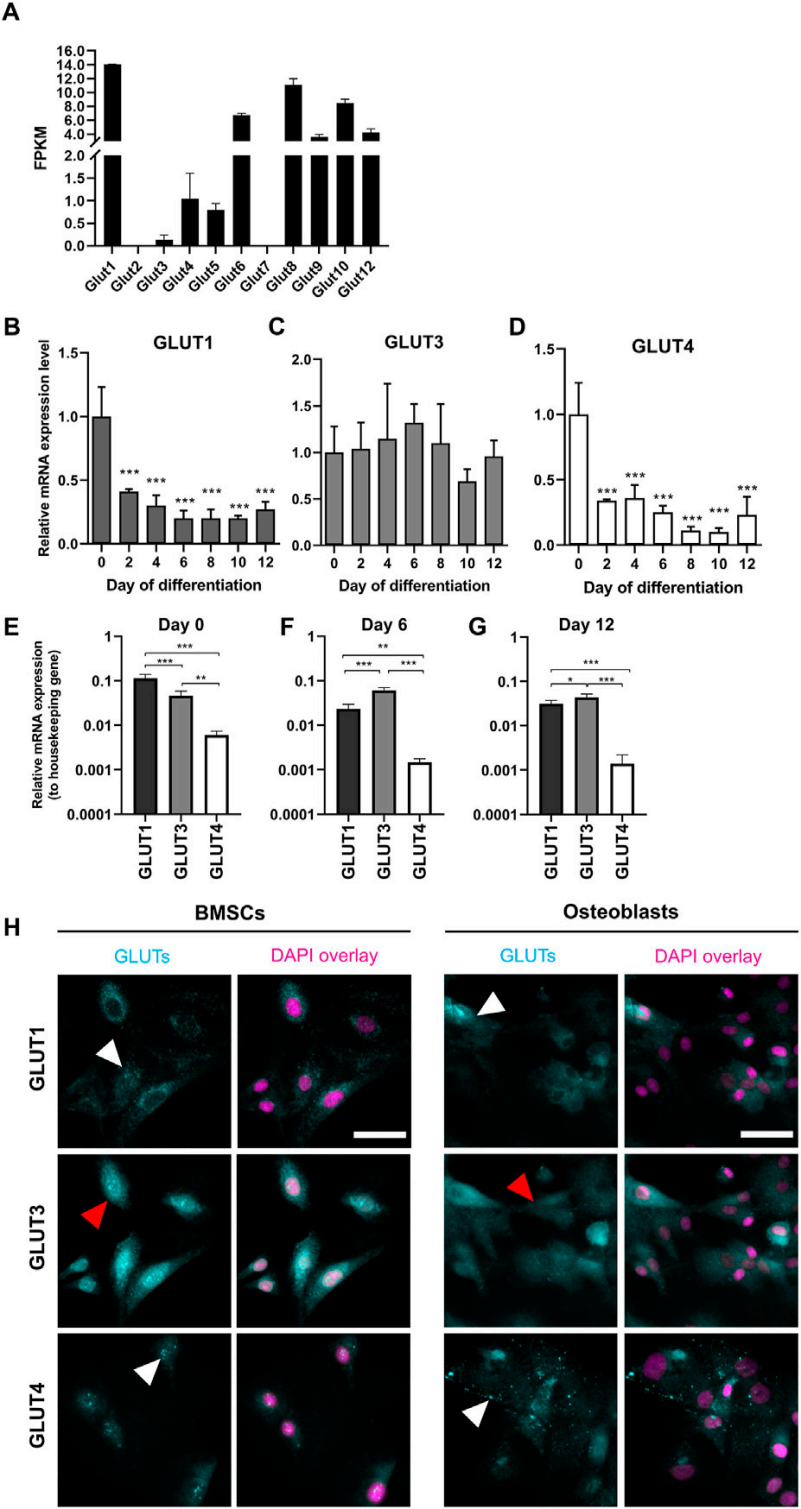
Expression pattern of GLUTs during osteoblast differentiation. Presence of GLUT transcripts in dataset of RNA-sequencing for day 2 osteoblasts **(A)**. Fragments per kilobase of transcript per millions reads mapped (FPKM) for each GLUT are shown. RNA-seq data cannot be used to quantitatively compare expression levels. GLUT1 **(B)** and GLUT4 **(D)** mRNA-expression levels decreased during osteoblast differentiation when compared to BMSCs (Day 0). GLUT3 **(C)** expression remained relatively stable. GLUT1 was the most expressed in BMSCs **(E)**, whereas GLUT3 was expressed in differentiated osteoblasts **(F,G)**. GLUT4 was the least expressed throughout the differentiation. Cyclophilin B was used to normalize the mRNA levels. Cultures were repeated 3 times and representative images are shown. When we stained GLUT-proteins (cyan) in BMSCs and day 6 osteoblasts, GLUT1 and GLUT4 appeared to be on intracellular vesicles (white arrows), whereas GLUT3 appeared to be more evenly distributed on cellular membrane (red arrows) **(H)**. Nucleus is shown with magenta, scalebar 50 μm. Note that GLUT1 and GLUT3 staining was performed as double-staining on same cells, and images are pseudo-coloured. *p*-values: *p < 0.05, **p < 0.01 and ***p < 0.001 (One way ANOVA).

The presence of GLUT1—12 transcripts in preosteoblasts was then determined by analyzing RNA-seq data. All other glucose transporters were detected, except for GLUT2 and GLUT7 which had no reads mapped (Figure 2A). GLUT11 was also absent, as rodents lack gene for GLUT11 (Scheepers et al., 2005). We then focused on class I glucose transporters which are all transporting glucose into mammalian cells, and evaluated the expression patterns of GLUT1, GLUT3, and GLUT4 in BMSCs and in differentiating osteoblasts. Expression level of GLUT1 significantly decreased already after 48 h of differentiation when compared to BMSCs (59%, *p* < 0.0001). In mature osteoblasts, the decrease was up to 80% (Figure 2B). Similarly to GLUT1, the expression of GLUT4 decreased during osteoblast differentiation. Expression decreased by 66% already after 48 h (*p* < 0.0001) and the expression levels continued to decrease by up to 90% later during differentiation (Figure 2D). In contrast, GLUT3 expression remained relatively stable during the entire 12-day differentiation period (Figure 2C). GLUT2 was not detected with qPCR (data not shown), supporting the outcome of RNA-seq results.

To determine which of the main glucose transporters, GLUT1, GLUT3 or GLUT4 are the most abundant isoforms at different stages of osteoblast differentiation, we compared the expression levels of each GLUT to a housekeeping gene (cyclophilin B). GLUT1 was found to be most abundant isoform in BMSCs (*p* < 0.0001, Figure 2E). Later during differentiation, at days 6 (Figure 2F) and 12 (Figure 2G), GLUT3 expression appeared to be higher when compared to GLUT1 (*p* < 0.0001 and *p* = 0.03, respectively). GLUT4 was the least abundant of these three isoforms throughout the differentiation of rat osteoblasts.

We then confirmed the protein expression of GLUT1, GLUT3, and GLUT4 in BMSCs and osteoblasts by immunostaining with standard imaging setup (Figure 2H). Both GLUT1 and GLUT4 proteins appeared to be abundant in cytoplasmic vesicles in both BMSCs and osteoblasts (white arrowheads), while GLUT3 protein was more evenly distributed in cellular membranes (red arrowheads). mRNA levels were not directly translated to observed protein levels. Despite dramatic downregulation observed in GLUT1 mRNA during differentiation, GLUT1 protein expression pattern was rather similar in both BMSCs and osteoblasts.

### Silencing of glucose transporters affects the proliferation of early osteoblasts

**FIGURE 3:**
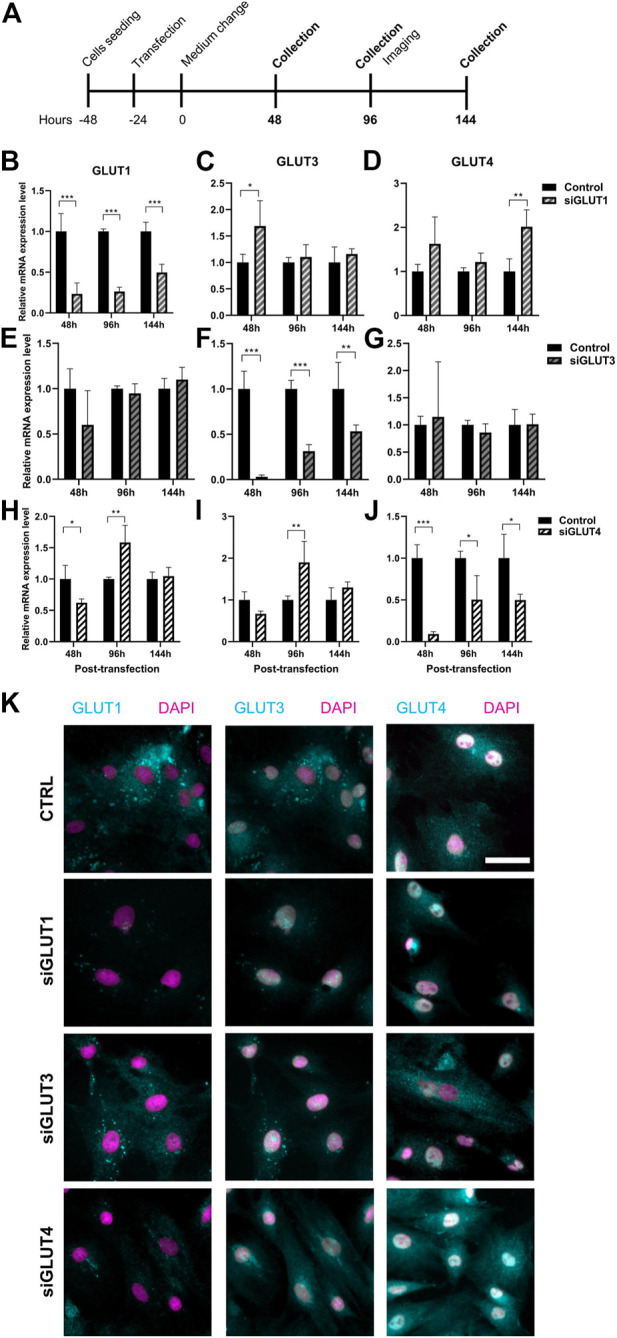
Silencing of GLUT1, GLUT3, or GLUT4. We then silenced GLUT1, GLUT3, or GLUT4 individually with siRNA-technology **(A)**. Cells were efficiently although transiently silenced using siRNA against GLUT1 (siGLUT1, B), GLUT3 (siGLUT3, F) or GLUT4 (siGLUT4, J). Universal scramble siRNA was used as a control. siRNAs were specific and no downregulation of GLUT1, GLUT3 and GLUT4 were observed **(B–J)**. mRNA expressions are compared against housekeeping gene (Cyclophilin B). Note that the *x*-axis are different. GLUT1, GLUT3 and GLUT4 protein expression in osteoblasts at 96 hrs post-transfection **(K)**. GLUTs are shown with cyan and nucleus with magenta, scalebar 50 μm. Note that GLUT1 and GLUT3 staining was performed as double-staining on same cells, and images are pseudo-coloured. *p*-values: *p < 0.05, **p < 0.01, ***p < 0.001 (One way ANOVA).

To study the relevance of GLUT1, GLUT3, and GLUT4 for BMSCs and osteoblasts, we transiently silenced GLUT1, GLUT3 or GLUT4 with specific siRNA constructs (Figure 3A). Scramble construct was used as a control. Transfection of primary BMSCs was successful (Supplementary Figure S1) and an efficient silencing of each GLUT was obtained. After 48 h of silencing, the expression of GLUT1 was suppressed by 77% (Figure 3B), GLUT3 by 97% (Figure 3F) and GLUT4 by 91% (Figure 3J). Six days after transfection, downregulation of approximately 50% was still maintained.

Silencing was highly specific for each of the GLUT mRNAs (Figures 3B–J). Downregulation of GLUT1 resulted in moderate increase in the expression of GLUT3 48 h post-transfection (*p* = 0.04) but not at later time points (Figure 3C). The expression of GLUT4 was also increased at 144 h (Figure 3D). When we downregulated GLUT4 expression, GLUT1 expression levels was reduced at 48 h but later both GLUT1 and GLUT3 levels increased 96 h post-transfection (Figures 3H,I). Downregulation of GLUT3 did not have any detectable effects on the expression levels of either GLUT1 (Figure 3E) or GLUT4 (Figure 3G). At protein level, GLUT1 seemed to be less abundant in all three silencing experiments, while GLUT3 and GLUT4 protein levels appeared to be unaffected at this time point (96 h) (Figure 3K). Interestingly, we noticed a spatial redistribution of GLUT3 from vesicular compartment upon silencing.

**FIGURE 4:**
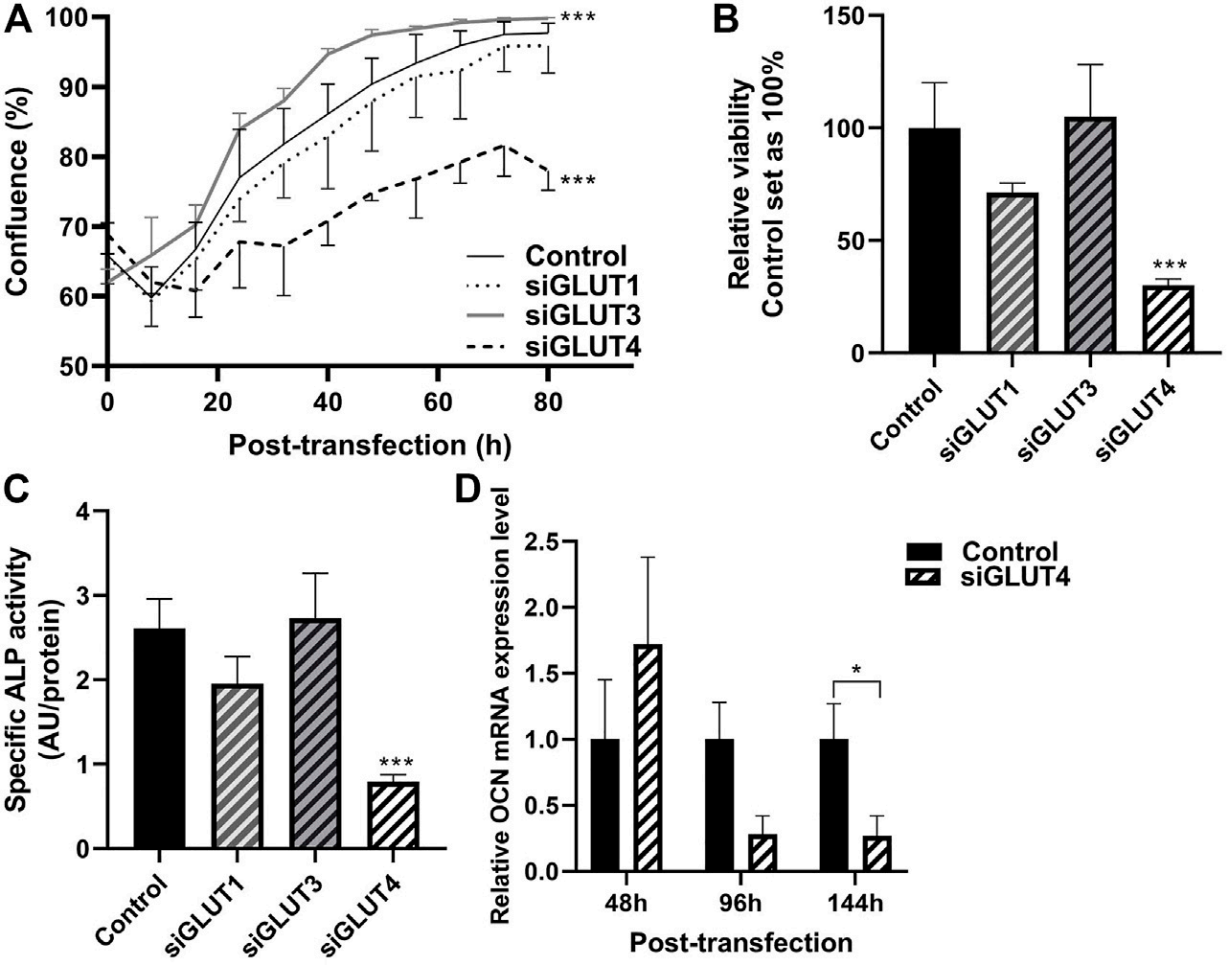
Silencing of GLUTs have different effects on proliferation and differentiation. Proliferation of silenced cells was studied by measuring cell confluence with using the IncuCyte real-time imaging system. After transfection, medium was changed to OM medium and cells were imaged up to 92 h (80 h shown) **(A)**. Statistical significances were calculated by calculating area under the curve and comparison was made with unpaired *t*-test. At the end point, viability of the cells was determined with alamarBlue–reagent **(B)**, differentiation was assessed with determining relative ALP activity to protein concentration **(C)** and relative osteocalcin expression compared to housekeeping gene **(D)**. Statistical difference was determined with Mann Whitney U or one-way ANOVA *p* values: *p < 0.05, ***p < 0.001.

We then assessed the growth properties of GLUT-silenced osteoblasts by IncuCyte imaging. Silencing of GLUT3 increased the proliferation and growth of osteoblasts (*p* < 0.0001), and corresponding confluency was reached approximately 24 h earlier than in scramble-treated control cells (Figure 4A). In contrast, silencing of GLUT4 resulted in a significant decrease (*p* < 0.0001) in cell proliferation rate and cells with low GLUT4 levels failed to reach confluency during the observation period of the experiment. Silencing of GLUT1 did not have detectable effect in cell proliferation rate. We also analyzed the viability of cells at the end of the culture, 5 days post transfection. Cell viability was significantly decreased when GLUT4 (*p* = 0.0001) was silenced compared to the scramble control. Silencing of GLUT1 or GLUT3 did not have an effect on cell viability at the endpoint, when cells had already reached 100% confluency (Figure 4B).

We then evaluated whether downregulation of any GLUTs has an effect on osteoblast differentiation. Silencing of GLUT4 significantly decreased the activity of ALP protein compared to control (Figure 4C) (70%, *p* < 0.0001). Expression of late osteoblast marker, OCN, was significantly decreased by 70% in mature osteoblasts, at day 6 of differentiation (Figure 4D) (*p* = 0.029). mRNA levels of ALP and Runx2 remained relatively unchanged after GLUT4 silencing (*p* > 0.05, data not shown). Silencing of GLUT1 or GLUT3 had no major effects on mRNA levels of Runx2, ALP or OCN (data not shown) or ALP activity (Figure 4C).

### RNA sequencing reveals global changes in metabolism and cellular organization

**FIGURE 5:**
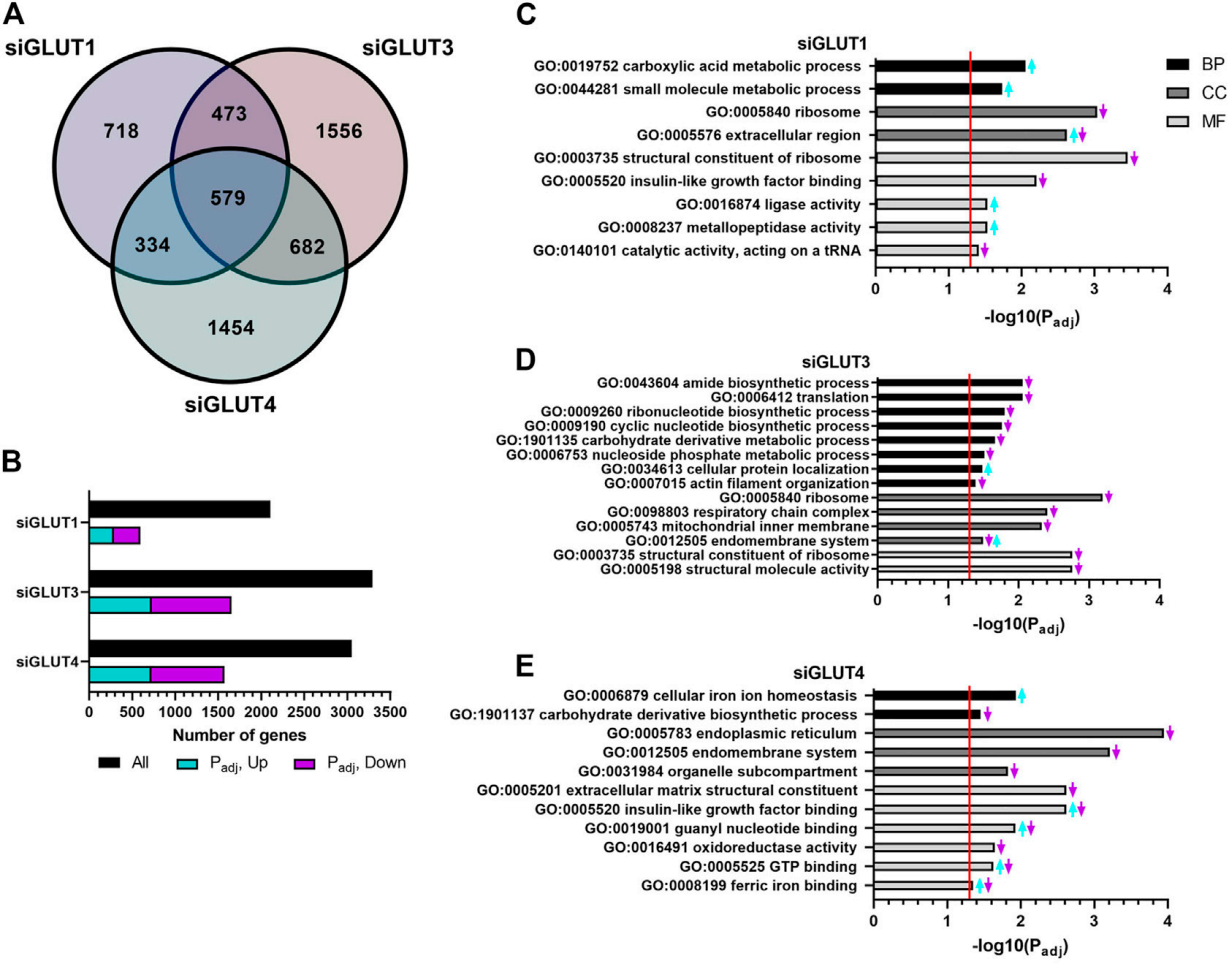
RNA sequencing of silenced cells reveals unique changes in gene expression. GLUT-silenced cells were compared to scramble control at 48 h post transfection and number of differentially expressed (DE) genes in each group are shown in Venn diagram **(A)**. Number of up- and down-regulated DE genes after *p*-value adjustment **(B)**. Functional analysis of DE genes (p_adj_ < 0.05) in GLUT1 **(C)**, GLUT3 **(D)**, and GLUT4 **(E)** silenced cells was done by common pathway enrichment analysis referencing to Gene Ontology database.

RNA sequencing analysis revealed global changes in gene expression caused by silencing of GLUTs. The number of differentially expressed (DE, *p* < 0.05) genes against scramble control for each siRNA treatment are presented in Figures 5A,B. Silencing of GLUT1 resulted in 2104 DE genes of which 718 were unique (Figure 5A). When adjusted for multiple comparison, 595 genes remained differentially expressed and of those, 48% (288) were up and 52% (307) down regulated (Figure 5B). Silencing of GLUT3 or GLUT4 resulted in greater changes in transcriptome. Silencing of GLUT3 resulted in 3290 DE genes of which 1556 were unique (Figure 5A). When further adjusted for multiple comparison, 1653 DE genes remained significant, and of those 44% (727) were up and 56% (926) were down regulated (Figure 5B). Silencing of GLUT4 resulted in 3049 DE genes of which 1454 were unique (Figure 5A), and when adjusted for multiple comparison 1573 genes were differentially expressed. Of those, 46% (723) were up and 54% (850) were down regulated (Figure 5B). Overall, silencing of GLUT1 resulted in smaller global change in the gene expression compared to silencing of either GLUT3 or GLUT4 which both had rather similar amounts of total and unique changes.

DE genes were used for common pathway enrichment analysis utilizing the Gene Ontology annotations (Figures 5C–E) to further understand GLUT-related pathways in osteoblasts. Figure 5 has been made to best represent the overall changes in the most enriched pathways that were statistically significant. Original, unaltered list of the top 10 most significantly changed pathways can be found in the Supplementary Figure S2. Enriched pathways are categorized by biological processes (BPs), cellular components (CCs), and molecular functions (MFs).

Pathways enriched in GLUT1-silenced preosteoblasts contained e.g. upregulation of carboxylic acid and other small molecule metabolism (e.g., Pyruvate kinase, *Pkm;* guanylate cyclase soluble subunit 1, *Gucy1b3;* Glutamine dehydroxylase 1, *Glud1;* cationic amino acid transporter*, Slc7a1*) (Figure 5C). Downregulated pathways included those of ribosome and its structural components. Further, upregulation of extracellular matrix production (e.g., decorin, *Dcn*; lumican, *Lum*; versican, *Vcan*; hyaluronan synthase 2, *Has2*) and metallopeptidase activity (e.g., several members of the ADAMTS-family) was observed.

More pathways were altered in GLUT3-silenced preosteoblasts. Biosynthetic pathways of ribonucleotides, cyclic nucleotides and amides, as well as protein translation and carbohydrate metabolism were all downregulated, suggesting multitude of changes in cellular metabolism. Low biosynthetic activity was supported by downregulation of ribosomal components. In line with this, pathways related to mitochondrial inner membrane proteins were suppressed, including components of respiratory chain complex (e.g., subunits of NADH dehydrogenase *Ndufa6, Ndufa13, Ndufa11,* and *Ndufb6*; subunits of cytochrome c oxidase *Cox6a1, Cox8a*; and ATP synthase subunits *Atp5e, Atp5j, Atp5g1,* and *Atp5l*). Interestingly, pathways involved in actin organization (including α1, α2, and *β* actins), endomembrane pathways and cellular protein localization were also altered (Figure 5D).

In response to GLUT4-silencing (Figure 5E), metabolic pathways involved in eg. carbohydrate derivative biosynthesis and oxidoreductase activity (e.g., HMG-CoA reductase, *Hmgcr*; isocitric dehydrogenase, *Idh1*; hydroxyacyl-CoA dehydrogenase*, Hadh*) were suppressed. Several pathways related to cellular membrane compartments were downregulated, such as components of endoplasmic reticulum, endomembrane system and organelle compartmentalization. Changes were also seen in cell signaling, including pathways related to binding of IGF (*Igfbp3, −4, −6,* and *−7*) as well as to guanyl nucleotide and GTP (e.g., guanylate cyclase 1, *Gucy1a3* and *Gucy1b3*). An upregulation of transcripts involved in cellular iron homeostasis and ferric ion binding (e.g., ferritin, *Ftl1*) was observed in GLUT4-silenced preosteoblasts. Of note, pathways involved in extracellular matrix (ECM) structural components, such as genes related to collagen synthesis (e.g., prolyl 4-hyroxylase*, P4ha1, -a3, -b*) were downregulated, suggesting impaired matrix production in response to GLUT4-silencing.

## Discussion

In this study, we found that class I glucose transporters GLUT1, GLUT3, and GLUT4 had distinct expression patterns during osteoblast differentiation. GLUT1 was abundant in BMSCs but downregulated along osteoblast differentiation, and similar trend was observed for GLUT4. The expression of GLUT3 remained unchanged. Live-cell imaging and RNA-seq analysis of GLUT-silenced osteoblasts revealed unique effects for GLUT1, −3 and −4 preosteoblasts.

Skeleton is a site of significant glucose uptake and utilization. *In vivo* animal studies have revealed glucose uptake in several parts of mouse skeleton, including calvaria, vertebra and tibia, using 18F-FDG-PET/CT imaging (Zoch et al., 2016). In long bones, uptake was predominantly observed in epiphyseal and metaphyseal regions, which contain trabecular bone and high remodeling activity. We have also detected and analyzed glucose uptake in bone marrow compartment (Ojala et al., 2020). However, resolution of *in vivo* imaging techniques does not allow detailed analysis at cellular level. We therefore silenced class I glucose transporters in primary rat osteoblasts in physiological conditions (5.5 mM glucose) to investigate the role of each GLUT for osteoblast biology.

GLUT1 remains one of the most studied glucose transporter and bone-forming osteoblasts are no exception (Zoidis et al., 2011; Wei et al., 2015; Li et al., 2016; Kanazawa et al., 2018; Chen et al., 2019). GLUT1 is expressed in most tissues and speculated to be responsible for basal glucose uptake (Pragallapati and Manyam, 2019). We found, that GLUT1 was the most abundantly expressed glucose transporter in BMSCs, but the expression levels dramatically decreased during osteoblast differentiation. GLUT1 is abundant in fetal tissues and stem cells (Maurer et al., 2006; Mueckler and Thorens, 2013), which could partially explain high expression observed in undifferentiated BMSCs. However, GLUT1 did not appear to be indispensable for BMSCs and preosteoblasts, as efficient (>80%) and specific silencing had no major effects on osteoblast proliferation, differentiation or morphology. Only modest upregulation of GLUT4 expression levels was observed, suggesting partial compensation for reduced GLUT1. RNA-seq analysis of GLUT1-silenced osteoblasts revealed less profound changes in gene expression, when compared to GLUT3-or GLUT4-silenced cells, supporting less important role in osteoblast biology. Silencing does not result in complete ablation of the protein and remaining protein levels, although suppressed, may be sufficient to maintain osteoblasts functional. However, GLUT1 suppression resulted in upregulation of small molecule metabolic pathways and increased production of ECM components, suggesting alternative bioenergetics and commitment to osteoblast lineage, respectively. Wei et al. (2015) have shown that GLUT1 regulates Runx2 post-translationally and GLUT1-Runx2 interaction is required for osteoblast differentiation. We observed no changes in Runx2 mRNA levels when silencing GLUT1 (data not shown).

GLUT4 is responsible for insulin-stimulated glucose uptake. It is located on intracellular vesicles and translocated to plasma membrane upon insulin receptor stimulation (Mueckler and Thorens, 2013). We found that the expression levels of GLUT4 were markedly lower than the levels of GLUT1 in BMSCs and the levels further decreased during osteoblast differentiation. In contrast, Li et al. (2016) observed increased GLUT4 expression along osteoblasts differentiation, using neonatal calvarial murine osteoblasts. Variable expression patterns could be due to differences between mouse and rat models and may require further studies. Despite its relatively low abundance, GLUT4 appeared to be essential for proliferation of BMSCs and preosteoblasts, as GLUT4 silencing resulted in significant decrease in proliferation and differentiation. This is in line with findings by Li et al. (2016), who ablated GLUT4 in mature osteoblasts using Osteocalcin-Cre and observed reduced osteoblast proliferation and maturation *in vitro*. The molecular mechanisms linking GLUT4 downregulation to reduced cell proliferation and differentiation are not clear. Several bioenergetics pathways e.g., those related to carbohydrate biosynthesis, acetyl-CoA as well as IGF signaling were downregulated, providing one likely explanation for reduced cell proliferation and viability.

Compared to other main class 1 glucose transporters, GLUT3 has higher affinity to glucose and greater transport capacity (Simpson et al., 2008). It is abundant in cells that exhibit high glucose demand and reside in glucose-poor microenvironment, such as neurons, sperm (Simpson et al., 2008) and many cancer cells (Ziegler et al., 2020). Role of GLUT3 in osteoblasts has not been clearly established (Thomas et al., 1996; Mobasheri et al., 2002; Wei et al., 2015). We observed that GLUT3 was stably expressed throughout differentiation, both in BMSCs and differentiated osteoblasts, and localized on cellular membranes. Similar pattern has been previously reported in mouse calvarial osteoblasts (Li et al., 2016). Bone cells may require stable GLUT3 expression to ensure basal glucose uptake in low glucose conditions. Downregulation of GLUT3 resulted in suppression of mRNAs related to cellular metabolism and biosynthetic pathways. Against expectations, lack of GLUT3 accelerated the proliferation rate of BMSCs. Silencing of GLUT3 seemed to redistribute remaining intracellular GLUT3 reservoirs. This was supported by the upregulated protein localization pathways in our RNA-seq analysis. Changes in cellular distribution of GLUT3 has been reported for other cell types upon activation and high energy demand, such as in liver cells (Defries et al., 2016) and in platelets and neutrophils (Simpson et al., 2008).

RNA-seq revealed that osteoblasts were devoid of GLUT2 and GLUT7, whereas class III transporters, GLUT 6-, 8-, 10, and −12, were clearly detected in our RNA-seq, also after silencing of class I transporters (Supplementary Figure S3). Relatively little is known about class III transporters and their principal substrates and physiological roles remain unclear.

Strength of this study include specific and efficient silencing of each class I GLUT in primary cells, which allowed us to analyze their downstream pathways individually. We profiled GLUT expression patterns in detail at 2 day intervals to study sequential changes during differentiation of BMSCs to functional osteoblasts. Rats of same sex, age and genetic background were used to reduce inter-culture variation and we have successfully used this model previously (Arponen et al., 2020). Rat osteoblasts share many similarities with human osteoblasts, e.g., vitamin D response and organization of osteocalcin gene, which are both different in mouse osteoblasts (Booth et al., 2013). Our results and the existing data (Zoidis et al., 2011; Wei et al., 2015; Li et al., 2016) suggest some differences between mouse and rat models. However, we acknowledge that RNA sequencing reveals only transcriptional changes and it was performed only at one time point. Analysis point represents committed preosteoblasts and results cannot be extrapolated to later differentiation stages. We acknowledge that this study only examined glucose transporters at transcriptional levels and more specific assays to assess the glucose uptake would be ideal. Protein levels are regulated at multiple levels, such as *via* protein half-life and microRNAs, and functional GLUT proteins may be present despite lack of *de novo* GLUT mRNA synthesis. Normoglycemic conditions were used to minimize the effects that environmental glucose may have on glucose transporters, but it would be interesting to challenge the cells with either hypo- or hyperglycemia. Finally, we only evaluated the machinery for glucose uptake. Osteoblasts also utilize other substrates as energy resources, e.g., glutamine has been found important for BMSCs (Karner et al., 2015) chondrocytes (Stegen et al., 2020) and osteoprogenitors (Stegen et al., 2021), among others.

In summary, GLUT1, −3 and −4 may all contribute to glucose uptake in differentiating osteoblasts. GLUT1 appears to be most abundant in early proliferating precursors, while stable and uniform expression of GLUT3 suggest also a role for this high-affinity transporter. GLUT4 expression was required for proper osteoblast proliferation and differentiation and impaired GLUT4 pathway will have negative effects on bone formation. Presence of other GLUT family members may further contribute to fine-tuning of glucose uptake, but this calls for further studies. Overall, glucose uptake in osteoblast lineage appears to rely on several overlapping and complementing glucose uptake mechanisms to ensure sufficient energy for new bone formation.

## Data Availability

The datasets presented in this study can be found in online repositories. The names of the repository/repositories and accession number(s) can be found below: https://zenodo.org/record/7225310#.Y0_86nZByUk.
