# Supplementary material for: Glucose transporters GLUT1, GLUT3, and GLUT4 have different effects on osteoblast proliferation and metabolism
Source: Front Physiol. 2022 Nov 29;13:1035516. doi: 10.3389/fphys.2022.1035516 (PMC9744933; doi:10.3389/fphys.2022.1035516)
Supplement: Supplementary file 1 [file DataSheet1.pdf]

Supplementary Material

1 Cellular uptake of siRNA analogue.

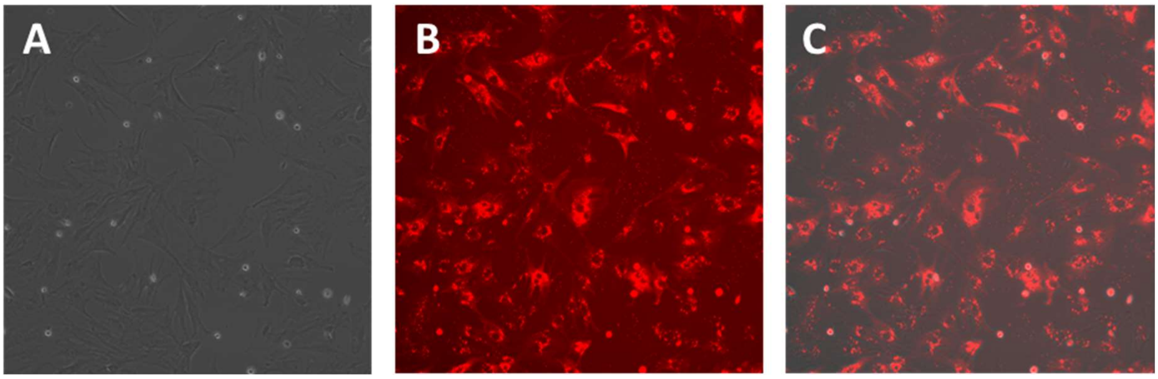

Supplementary Figure 1. Uptake of siRNA analogue was evident in BMSCs. BMSCs were transfected with 10 nM fluorescently-labeled siRNA analogue Trilencer-27 (Origene) in siTRAN2.0 for 18 hours. Cells were then imaged with EVOS M5000 Imaging System (Invitrogen, USA) to check siRNA delivery to the cells. Panel A shows BMSCs with a phase-contrast imaging. Red color on panel B indicates transfected cells. Overlay image shown in panel C confirms cellular uptake of siRNA analogue. Magnification 100x.

2 Supplementary table 1: Primer sequences

Table 1 Primer sequences

Symbol	Gene	Primer sequences (5'-3')	Length (bp)	Product (bp)	Tm (°C)
**Alpl**	NM_013059.1	F-TGCAGGATCGGAACGTCAAT	20	170	60
		R-GGGTCTTTCTCTTTCTCTGGCA	22		
**Bglap**	NM_013414.1	F-TGAGTCTGACAAAGCCTTCATGT	23	178	61
		R-GAAGCCAATGTGGTCCGCTA	20		
**Ppib**	NM_022536.1	F-ACCTGTAGGACGAGTGACCT	20	187	60
		R-GCTCTTTCCTCCTGTGCCAT	20		
**Runx2**	NM_053470.2	F-CGCCTCACAAACAACCACAG	20	173	60
		R-TGCAGCCTTAAATATTACTGCATGG	25		
**slc2a1**	NM_138827.1	F-GCCGCTTCATCATTGGAGTG	20	160	58
		R-GAGTCTAAGCCGAACACCTGG	21		
**slc2a2**	NM_012879.2	F-CCGGCACATGCTCTCATCAT	20	182	58
		R-CTGAGGCCAGCAATCTGACTA	21		
**slc2a3**	NM_017102.2	F-GATCCTTGTGGCTCAGGTCT	20	180	58
		R-ATCTCCGTCGCTTGGTCTTC	20		
**slc2a4**	NM_012751.1	F-CGCGGCCTCCTATGAGATAC	20	188	58
		R-ACTCCAAACCCAACACCTGG	20		

3 Results of RNA-seq analysis

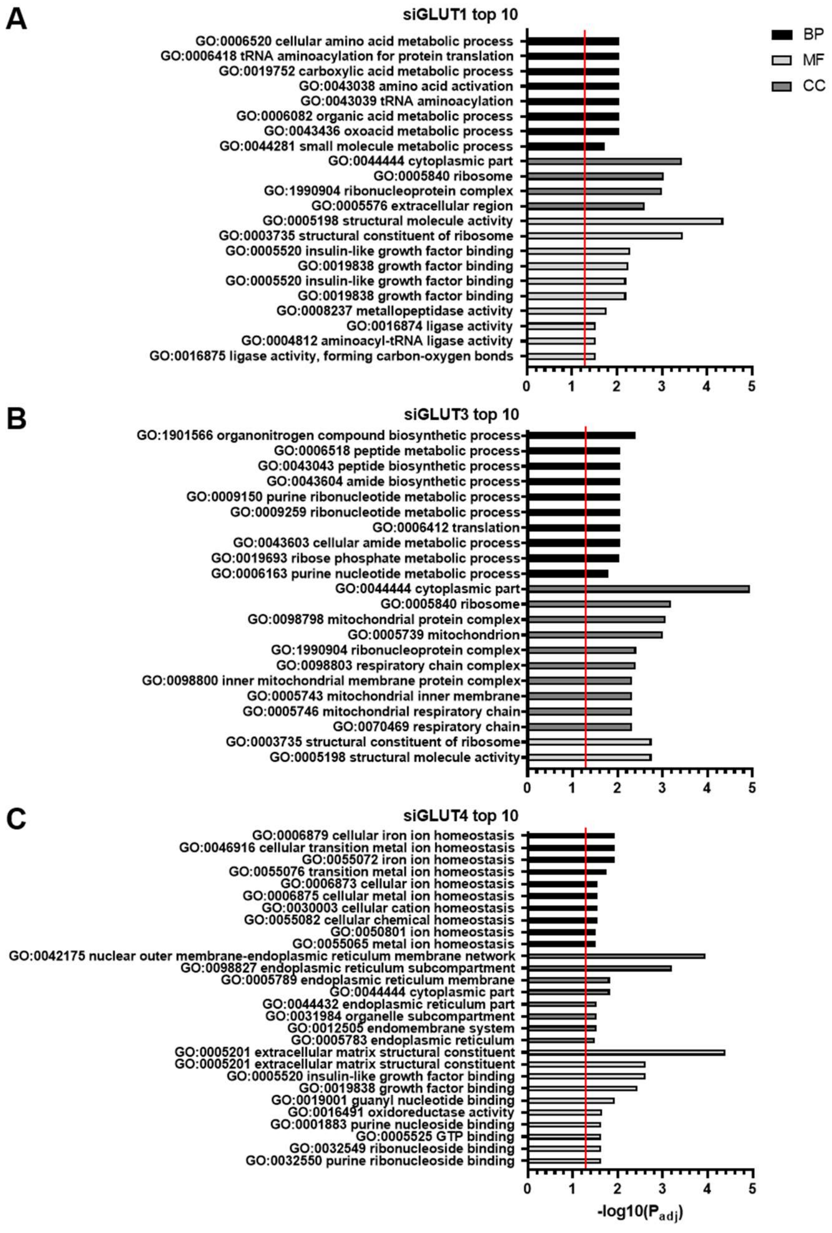

Supplementary Figure 2 Top 10 most significantly changed pathways due to silencing of either GLUT1 (A), GLUT3 (B) and GLUT4 (C). DE genes were used for common pathway enrichment analysis utilizing the Gene Ontology annotations. Enriched pathways are categorized by biological processes (BPs), cellular components (CCs), and molecular functions (MFs)

4 Transcriptome of GLUT family when silencing class I glucose transporters

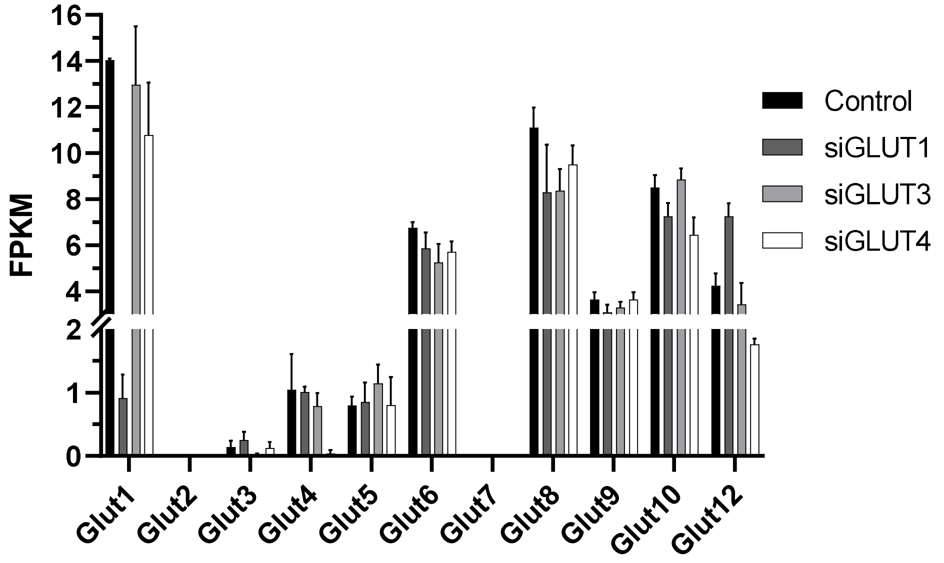

Supplementary Figure 3. Silencing of GLUT1 (dark grey), GLUT3 (grey) and GLUT4 (white) was specific and resulted in minor changes in transcription of other members of GLUT-family. Data is presented as fragments per kilobase of transcript per millions reads mapped (FPKM).
